# Talker-Specific Generalization of Pragmatic Inferences based on Under- and Over-Informative Prenominal Adjective Use

**DOI:** 10.3389/fpsyg.2015.02035

**Published:** 2016-01-20

**Authors:** Amanda Pogue, Chigusa Kurumada, Michael K. Tanenhaus

**Affiliations:** ^1^Department of Brain and Cognitive Sciences, University of RochesterRochester, NY, USA; ^2^Department of Linguistics, University of RochesterRochester, NY, USA

**Keywords:** sentence processing, adaptation, generalization, pragmatics, informativity, referential expressions

## Abstract

According to Grice’s (1975) Maxim of Quantity, rational talkers formulate their utterances to be as economical as possible while conveying all necessary information. Naturally produced referential expressions, however, often contain more or less information than what is predicted to be optimal given a rational speaker model. How do listeners cope with these variations in the linguistic input? We argue that listeners navigate the variability in referential resolution by calibrating their expectations for the amount of linguistic signal to be expended for a certain meaning and by doing so in a context- or a talker-specific manner. Focusing on talker-specificity, we present four experiments. We first establish that speakers will generalize information from a single pair of adjectives to unseen adjectives in a speaker-specific manner (Experiment 1). Initially focusing on exposure to underspecified utterances, Experiment 2 examines: (a) the dimension of generalization; (b) effects of the strength of the evidence (implicit or explicit); and (c) individual differences in dimensions of generalization. Experiments 3 and 4 ask parallel questions for exposure to over-specified utterances, where we predict more conservative generalization because, in spontaneous utterances, talkers are more likely to over-modify than under-modify.

## Introduction

A key feature of human language is that there are many-to-many mappings between referents and linguistic expressions. A pet dog can be referred to by many expressions (e.g., *the dog*, *Charlie*, *he*, or *my friend*) whereas the expression *the dog* can be used to refer to a real dog, a toy dog, or a contemptible person. Referential expressions can also be made arbitrarily long (e.g., *the big dog*, *the big brown dog*, *the big brown furry dog, etc.*). One long-standing issue in psycholinguistic research is how language users map a referential expression onto an intended referent with the speed and accuracy evidenced in real time language use (e.g., Altmann and Steedman, 1988; Eberhard et al., l995; Allopenna et al., 1998; Arnold et al., 2000; Brown-Schmidt and Hanna, 2011).

One influential hypothesis is that listeners cope with this mapping problem by assuming that speakers behave rationally, formulating their utterances to be as economical as possible while conveying all necessary information (Grice, 1975). Hereafter, we call this the rational-speaker model. For example, a rational speaker is more likely to use a pre-nominal scalar adjective (e.g., *the big dog*) when there is a complement (contrast) set of referents of the same semantic type (e.g., a big and one or more small dogs) in the same context (Sedivy et al., 1999; Davies and Katsos, 2009). By assuming a rational model of the speaker, listeners can make predictions about the referring expression that maximize the informativity of a linguistic element, where informativity is defined as the amount of uncertainty that is reduced by the element given the set of plausible referents in the current referential domain (Frank and Goodman, 2012). Frank and Goodman (2012) tested the informativity hypothesis using a simple language game. With three geometrical shapes with two shape features and two colors (e.g., a blue square, a blue circle, and a green square), comprehenders were asked to pick the referent that best matched a single word description (e.g., *blue* or *square*). A rational language user model predicts that when given *blue* participants should most frequently choose the blue square rather than the blue circle. This is because if the talker had meant the blue circle, she should have used the more informative (unambiguous) description *circle*. The results confirmed this prediction.

Real-time processing of prenominal adjectives is also influenced by the assumption that the speaker is formulating her utterances to efficiently pick out a referent given contextually salient contrast sets. In a visual world study (Tanenhaus et al., 1995), Sedivy et al. (1999) used spoken instructions such as “Pick up the tall glass” in a visual workspace with a tall glass and a short glass (which form a contrast set), a tall pitcher and an unrelated object (e.g., a key). A rational speaker would use the adjective “tall” to refer to the glass, which is a member of a contrast set, and not the tall pitcher. If listeners use the context and make this inference in real-time, as they hear the adjective, “tall,” they should begin to look at the tall glass. This is just the result reported by Sedivy et al. (also see Hanna et al., 2003; Heller et al., 2008; Wolter et al., 2011).

Although these results are consistent with a rational model of reference generation and understanding, some researchers have questioned whether a rational model will scale up to account for interlocutors’ behavior in everyday language use. Spontaneously produced referential expressions often include information that would be superfluous under the assumption that the speaker should only provide necessary and sufficient information. For example, spontaneous utterances often contain prenominal modifiers that are not necessary for identifying a unique referent (Deutsch and Pechmann, 1982; Sonnenschein, 1984; Pechmann, 1989; Belke, 2006; Engelhardt et al., 2006; see also Koolen et al., 2011). For instance, 30% of speakers used superfluous adjectives in a production study in Engelhardt et al. (2006) and 50% in Nadig and Sedivy (2002).

Conversely, interlocutors frequently under-specify in highly specific circumstances. In Brown-Schmidt and Tanenhaus (2008), interlocutors were tasked with rearranging blocks on puzzle boards. Areas in the workspace were divided into sub-regions. More than 50% of the referential expressions were underspecified with respect to potential referents in the relevant sub-region. Nonetheless, these utterances were seamlessly interpreted by the listener. Analyses showed that underspecified utterances only occurred when the alternatives were unlikely to be the intended referent given the local task constraints. For example a speaker might say, “put it above the red block,” when there were two red blocks but only one had a free space above it.

In sum, in relatively simple situations, like those typically examined in psycholinguistic studies, talkers often over-specify. In contrast, in more complex situations with richly structured discourse context, talkers frequently under-specify. For purposes of the present work, we will be focusing on situations where over-specification in the form of “redundant” prenominal adjectives is quite common and under-specification is relatively infrequent.

How can we reconcile the ubiquitous over-specification in these situations with the evidence that listeners seem to assume that a prenominal adjective is included to form a maximally informative utterance with respect to the context? One possibility is that the rational assumption is only one of many relevant factors that the talker and the listener take into account, rather than a strong determinant of reference generation and understanding. For example, in an interactive communication game, Engelhardt et al. (2006) reported comprehenders’ asymmetrical reactions to over- and under-modifying expressions. Comprehenders judged an ambiguous, under-specifying, expression in the presence of more than one plausible candidate to be less than optimal. However, they did not seem to draw additional inferences from superfluous, over-specifying, descriptors (see also Davies and Katsos, 2010, for evidence of asymmetrical penalization of over- vs. under-modified expressions by adults and children in a non-interactive task). Based on these asymmetrical findings between under-and over-informative utterances, Engelhardt et al. (2006, p. 572) concluded, “people are only moderately Gricean.”

Before adopting this conclusion, there is another approach that we believe is worth exploring. This approach is motivated, in part, by work that reevaluates what it means to be *rational* in decision-making. In a seminal line of research, Tversky and Kahneman (1974) documented ways in which human agents systematically deviate from the rational models widely assumed within economics. They proposed that people rely on heuristics, such as availability, similarity and representativeness that can result in fallacies leading to non-rational, or non-logical, decision-making under many circumstances. One such case is the “conjunction fallacy” where given a scenario about, Linda, a college-educated woman who cares deeply about social issues, participants will rate the likelihood the Linda is *bank teller and a feminist* as greater than the likelihood that she is *a bank teller* (Tversky and Kahneman, 1983). This clearly violates a basic rule of logic and probability—a conjunction cannot contain more members than either of its conjuncts. These fallacies were therefore taken to suggest that human agents are not rational in their decision-making behaviors.

However, the same evidence can be viewed as consistent with the hypothesis that participants are behaving according to basic assumptions about the rationality of language users. One of the assumptions is relevance in information. In Grice’s terms, “Our talk exchanges do not normally consist of a succession of disconnected remarks, *and would not be rational if they did”* (Grice, 1975, p. 45, emphasis original). Based on this assumption, when the talker provides certain information (e.g*., Linda is a feminist*), the listener infers that she must have had a reason to do so with respect to the goal of achieving successful communication. Rationality, in this sense, manifests itself in the general tendency for language users to engage in goal-directed acts of communication even in the simple task used by Tversky and Kahneman rather than simply treating the scenario as an abstract logical problem (Hertwig and Gigerenzer, 1999; also see Oaksford and Chater, 2001 for a similar approach applied to other decision problems). Thus what might appear to be departures from rationality are in fact grounded in principled behaviors that overall lead to more successful communication^1^.

When we apply this perspective to reference generation and comprehension, it seems plausible that what we might view as departures from the rational-speaker model could, in fact, be fully consistent with a rational perspective. Let us assume that one of the most prominent goals of linguistic communication is to successfully convey intended messages and that this communication takes place through a noisy channel. It is essential, then, for the speaker to provide listeners with sufficient information while taking into account the likely possibility that some information will be lost due to noise in the production and comprehension systems (e.g., Aylett and Turk, 2004; Levy and Jaeger, 2007; Jaeger, 2010; Gibson et al., 2013). In particular, early in an interaction, interlocutors are likely to have uncertainty about the relevant context that bears on the current interaction and the degree to which they have shared goals and experience, etc. There is also variability in how well-different talkers and listeners take into account each other’s perspective, individual differences along dimensions, such as spatial ability, and differences in speech style (e.g., the degree to which abrupt utterances are considered impolite). Given these considerations, it can be *rational* to provide more information than what is minimally required, rather than trying to estimate what degree of specification is optimal. This tendency is likely strengthened in non-interactive tasks in which a talker cannot negotiate with her listener during the interaction.

Indeed, there is evidence that listeners can take into account such communicative considerations from the speaker’s perspective. Davies and Katsos (2009) proposed that the higher tolerance for over-informative expressions in Engelhardt et al. (2006) arises because these expressions can plausibly be attributable to communicative reasons (e.g., an extra effort for avoiding ambiguity). When the redundancy is unlikely to benefit communication, comprehenders found the over-informative utterances to be sub-optimal just as they do for under-informative utterances. Davies and Katsos’ (2009) results suggest that listeners do not simply judge whether an expression is over-informative given a referent, but they reason about why the speaker produced an additional element with respect to the goal of successful communication. As conversation unfolds and as interlocutors have an increasingly coordinated construal of common ground, expectations for referring expressions are also tightened in a talker- and context-specific manner (Metzing and Brennan, 2003; Brown-Schmidt et al., 2015; Kronmüller and Barr, 2015). As a result, what might appear to be an ambiguous referring expression becomes fully informative for interlocutors, allowing them to communicate more efficiently (Brown-Schmidt and Tanenhaus, 2008).

From this perspective, in contrast to Engelhardt et al.’s (2006) proposal, we hypothesize that variations found in referring expressions reflect rational principles for maximizing overall communicative success under uncertain conditions. We posit that listeners assume that talkers are generally Gricean, rather than only sometimes Gricean. Crucially, our framework assumes that (1) listeners expect talkers to vary in their choices of referential expressions and that (2) listeners constantly adapt their expectations about how much linguistic information a particular talker might provide to convey a particular referential intention. This allows listeners to navigate the variability in referring expressions to arrive at the intended referent.

As a first step in developing this approach, the current paper tests whether and, if so, how listeners adapt their referential expectations in simple communicative contexts similar to those used in many other psycholinguistic studies discussed above. In particular, we ask whether listeners adapt their expectations in a talker-specific fashion. This question is motivated by Grodner and Sedivy’s (2011) demonstration that listeners discount linguistic evidence for contrastive inference when they are told that the speaker has an impairment “that causes social and linguistic problems.” When receiving such a top–down instruction, listeners no longer interpret prenominal modifiers produced by the given talker as a meaningful cue to a contextual contrast (cf. Sedivy et al., 1999). With such a case of pragmatic impairment and a strictly rational model as two extreme ends of a continuum, talkers will often vary in terms of how much information they typically include in an utterance. Some talkers will be prone to provide additional descriptors while others’ utterances are more succinct. Each talker, however, is likely to be relatively consistent. To the extent that these assumptions hold, flexibly adapting an expected form of an utterance for a given talker will prevent listeners from going astray when they encounter more or less information than what is *a priori* expected.

To test this hypothesis, we created an experimental paradigm with an Exposure Phase and a Generalization Phase. In the Exposure Phase the input from one of two speakers deviates from what is expected based on the rational speaker model. Specifically, that speaker does or does not use a scalar adjective (e.g., big/small) that would be necessary for singling out a referent, or if used, would provide redundant information (under- and over-modifying speakers given the rational model). We then examine in a Generalization Phase whether listeners derive different referential expectations for these two speakers (i.e., talker-specific expectation adaptation). In addition, we present a previously unseen set of adjectives in the Generalization Phase to examine the robustness of the adaptation process. We hypothesized that rational listeners would generalize from their experience, resulting in more accurate expectations for a wider range of linguistic expressions than those for which they have direct evidence. For example, upon observing utterances with referring expressions from a talker who provides over-specified expressions along one dimension (e.g., big/small), a listener might infer that that talker would also be more likely to over-specify along other dimensions (e.g., skinny/wide). (In Experiments 3 and 4, we provide a direct test of this prediction with adult native speakers of English.) We thus examine listeners’ adaptation of referential expectations for uses of observed and unobserved adjectives.

One important factor that influences patterns of generalization is listeners’ prior beliefs about how talkers might vary in their reference generation. For example, an instance of a seemingly over-specifying adjective can be compatible with at least two hypotheses: (1) the talker is incapable of making an optimally informative utterance (informativity-based generalization), or (2) the talker prefers to produce a longer utterance (length/form-based generalization). Also, listeners need to determine if the over-specification is confined to (1) the particular type of adjective, (2) adjectives in general, or (3) any form of modification. Moreover, one episode of sub-optimal language use could be indicative of the talker’s overall pragmatic ability or it could be a random production error, in which case it would have little predictive power about future input. To avoid over- and under-generalization, rational listeners must evaluate the observed evidence against their prior beliefs to estimate how reliably it conveys information about the pragmatic competence of the talker (Xu and Tenenbaum, 2007; for a theoretical discussion on effects of prior beliefs in phoneme adaptation and generalization see Kleinschmidt and Jaeger, 2015). Based on this assumption, we predict a critical difference in how listeners generalize evidence of under- and over-specified utterances. Given the prevalent over-modification observed in natural discourse, a single instance of a redundant adjective use provides less reliable evidence that the speaker would be non-optimal in other domains of pragmatic language use compared to a single instance of under-specification. Therefore, we should see more conservative generalization (at the speaker-level) from evidence of over-specification compared to evidence of under-specification.

With the exception of pioneering work by Grodner and Sedivy (2011), talker- or context-specific adaptation and generalization of expectations have not thus far been studied extensively with respect to reference resolution (but see Arnold et al., 2007, for related discussion on comprehension of disfluencies). However, the importance of adaptation and generalization is increasingly appreciated in other domains of language processing. In particular, talker- and context-specific adaptation is crucial for comprehenders to navigate the problem of lack of invariance between the acoustic signal perceived and underlying linguistic categories such as phonemes. Some of this lack of invariance is due to random factors, such as errors in production and perception, but much is due to systematic factors, such as differences between speakers, dialects/accents, and speech conditions. A number of studies have demonstrated that listeners condition their perception of phonetic categories on talkers and their indexical features and learn to expect different acoustic features in the input for these different groups of talkers and different situations (e.g., Strand and Johnson, 1996; Niedzielski, 1999; Bradlow and Bent, 2008; Reinisch and Holt, 2014; for review see Drager, 2010; Kleinschmidt and Jaeger, 2015). Our framework shares a number of important properties with models developed to address the lack of invariance in speech perception. Most importantly, we view the problem of reference resolution as a form of systematic inference based on variable input in which listeners condition their inferences taking into account talker-specific information.

The remainder of the paper is structured as follows. We present results of four sets of experiments, in which we examine talker-specific generalization based on under-modified (Experiments 1 and 2), and over-modified (Experiments 3 and 4) utterances. We first establish that listeners will generalize information from a single pair of adjectives to unseen adjectives in a talker-specific manner based on observation of under-modified utterances (see Experiment 1: Talker-Specific Adaptation and Generalization Across Adjectives). We then tease apart two possible dimensions of talker-based generalization, which we call informativity-based and form-based generalization. A single observation of an under-modified utterance (e.g., “Click on the cup” in a presence of a big and a small cups) could be interpreted as evidence that the talker has a propensity to produce (1) under-informative expressions (i.e., informativity-based generalization) or (2) shorter expressions (form-based generalization). By introducing modified, yet under-informative utterances (e.g., “Click on the green cup” when the big and the small cups are both green), we demonstrate that whereas the generalization is primarily informativity-based some listeners more frequently made form-based generalizations (see Experiment 2A: Informativity-based vs. Form-based Generalization for Talker-Specific Adaptation). The preference for informativity-based generalization is magnified when the task is presented with an explicit instruction directing comprehenders’ attention to differences between the talkers (see Experiment 2B: Effects of Adding a more Explicit Cue – Focus on Naturalness), suggesting that construal of the task influences how listeners generalize from the evidence that they observe.

We then turn to exposure to over-modified utterances. Given the prevalence of such utterances in simple referring tasks, we predict more conservative generalization across adjective types compared to cases with under-modified utterances. The results suggest that the over-modified utterances are indeed unlikely to trigger informativity-based generalizations (see Experiment 3: Talker-Specific Adaptation with Over-Informative Evidence) although comprehenders do register that the two talkers’ utterances differ in length (see Ruling out an Alternative Explanation based on a Failure to Generalize overall for Over-Informative Utterances). This absence of informativity-based generalization persisted even when an extra manipulation highlighting the non-optimality of over-modifying utterances in referential communication was added (see Experiment 4: Drawing more Attention to the Fact that Over-Informative Information is not Helpful). In the General Discussion, we discuss an inference mechanism that provides a framework for explaining these different patterns of talker-specific generalizations of pragmatic information and suggests promising venues for future investigations.

## Experiment 1: Talker-Specific Adaptation and Generalization Across Adjectives

We first asked whether listeners would generalize information from observed to unobserved (new) adjectives in a talker-specific manner. Importantly, because listeners are unlikely to be given explicit, top–down information about pragmatic competence under most circumstances, we wanted to determine whether they would generalize without being explicitly told that the talker was pragmatically impaired as they were in Grodner and Sedivy (2011). In the Exposure Phase we introduced listeners to two talkers and tasked them with selecting the unique referent of the talker’s instruction from a set of four objects. The two talkers varied in their descriptions: only one talker used adjectives to pick out a unique referent (the modifying talker) while the other talker consistently used bare nouns (the non-modifying talker)^2^. In the Generalization Phase, we asked the listeners to guess which talker likely uttered transcribed instructions that were either modified (with new, or previously used adjectives) or unmodified. If listeners had generalized their assumptions about the talker’s adjective use, they should attribute both the observed and new adjective use to the modifying talker, and the unmodified instructions to the non-modifying talker.

### Methods

#### Participants

Thirty-two English-speaking adults residing in the USA were recruited online using the crowdsourcing platform Amazon Mechanical Turk (https://www.mturk.com/mturk/). Participants were compensated $1.00 for participating in the task^3^^,^^4^.

#### Materials

**FIGURE 1:**
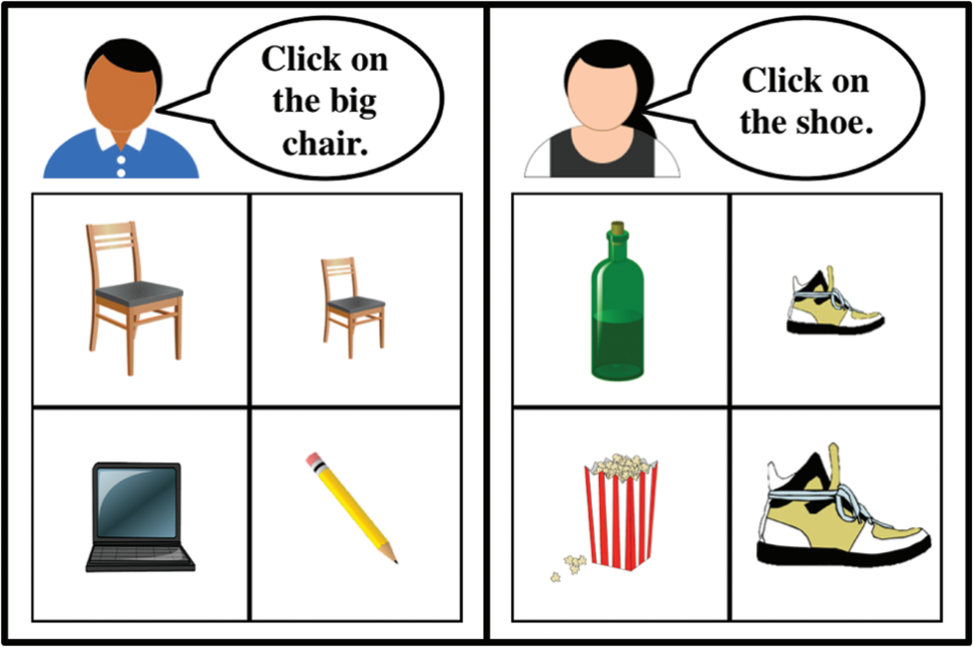
**An example of a trial (audio instructions are shown here in speech bubbles) with the modifying speaker (left) and the non-modifying speaker (right) from the Exposure Phase in Experiments 1 and 2**. Participants clicked on the image in the grid to respond.

We created 44 two-by-two grids of images (20 for exposure items and 24 for test items). Each grid has a contrast pair of images differing from each other in one dimension and distinguished with a scalar adjective (e.g., a big cake vs. a small cake as in **Figure 1**). The other two images were singletons.

Two native speakers of American English (one male and one female) recorded 10 instructions each for the 20 exposure items. All the instructions were of the form “Click on the ____” and the two speakers recorded three versions for each item: a bare noun (e.g., “Click on the cake”), and with the adjectives *big* (e.g., “Click on the big cake”) or *small* (e.g., “Click on the small cake”). 24 instructions were created for the Generalization Phase. One third of the modified instructions had the adjectives used in the Exposure Phase (four instructions each with *big* and *small*). The remaining two thirds of the modified instructions used new adjective pairs (four *tall*/*short*, four *skinny*/*wide*). Generalization instructions were presented as written scripts.

#### Procedure

In the *Exposure Phase*, participants were shown two-by-two grids of images. We provided a cover story that two naïve talkers had participated in a production task and produced instructions of the form “Click on the ___.” The job of current participants was to follow these instructions and select one picture by clicking on it. On 10 of the trials one of the speakers (the modifying talker) made a request using a prenominal adjective such as, “*Click on the big/small cake*” (five items with *big* and five items with *small*). On the remaining 10 trials the other talker (the non-modifying talker) produced instructions with bare nouns (e.g., *“Click on the cake”*). On each trial an avatar depicted which of the speakers the participant would hear on that trial (see **Figure 1** for an example of an Exposure Phase trial). The items were presented in a randomized order. The location of the target object, adjective (big vs. small), and gender of the modifying talker were counterbalanced across participants. Participants were instructed to make their best guess when they thought the speaker was unclear, or if they were uncertain. Participants were not given any feedback about their responses.

**FIGURE 2:**
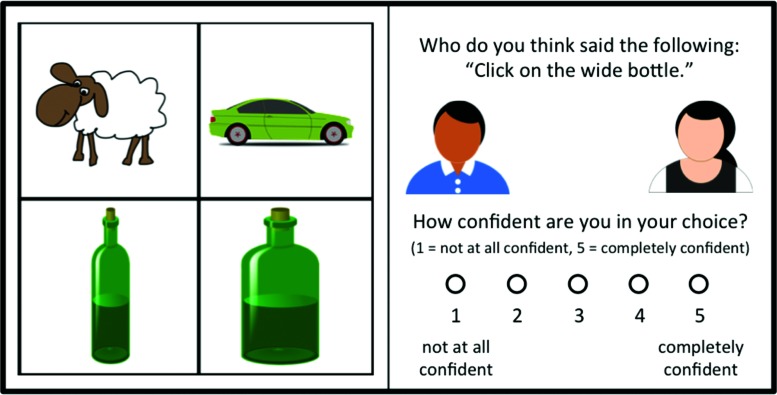
**In the Generalization Phase participants saw 2x2 image grids (left) above the transcribed instructions, avatars that represented the two speakers, and a confidence rating scale (right)**.

In the *Generalization Phase*, participants were told that they would read instructions that had been transcribed. Their task was to judge which of the two speakers was more likely to have produced the instruction and click on the corresponding avatar (**Figure 2**). 12 of the 24 instructions contained a modifying adjective. Four of the modified instructions contained the same adjectives as in the Exposure Phase (big/small); eight contained new scalar adjective pairs (two skinny/two wide, two tall/two short). On the remaining 12 trials the instructions were unmodified. These items were presented in a randomized order. The adjective-object pairing and type of instruction (modified vs. unmodified) was counterbalanced across participants. After making their selection participants were asked to rate how confident they were in their response on a five-point scale (1 = not at all confident, and 5 = completely confident).

### Results and Discussion

**FIGURE 3:**
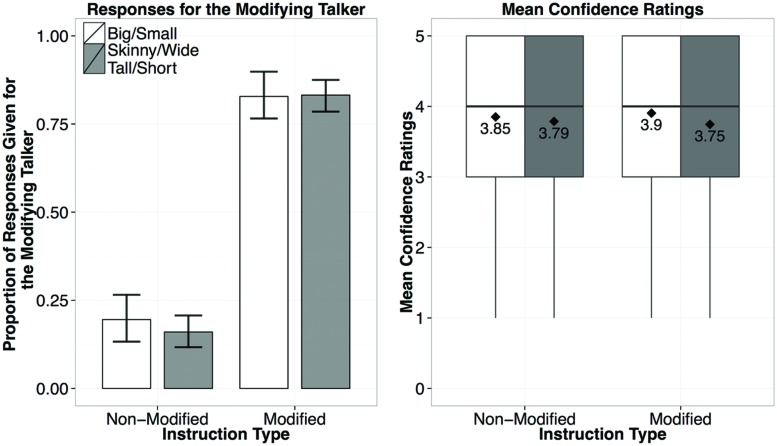
**Results from Experiment 1, showing the proportion of responses given for the modifying talker by Instruction Type (left), and the confidence ratings for responses by Instruction Type (right; diamond dots reflect the mean rating out of 5)**.

Choices in the Generalization Phase are plotted in **Figure 3**. Participants selected the modifying-speaker, who used *big/small* in the Exposure Phase, for the sentences with *big/small* (83%), and the non-modifying speaker in the unmodified trials (80%). Choice patterns for new adjectives were almost identical to those for exposure adjectives: 84 and 84% for skinny/wide, and 83 and 84% for tall/short. We constructed a mixed-model logistic regression of the responses given for the modifying speaker in the Generalization Phase with Adjective (exposed or new), and Instruction Type (modified or non-modified) as the fixed effects, and subject and item as the random effects^5^. We based our model on the recommendations for maximal Linear Mixed Effects Model (LMEM) as suggested by Barr et al. (2013) which takes into consideration the maximal random effects structure by including by-subject (Adjective and Instruction Type) and by-item (Instruction Type) random intercepts and slopes. We used the glmer function in lme4 in R, and specified a BOBYQA optimizer (Bates et al., 2015). As predicted, Instruction Type was the only significant predictor of whether participants would choose the modifying speaker (β = 5.84, *p* < 0.001). There were no reliable predictors of the confidence ratings (*p*s = 1), indicating that participants were equally certain (modified mean = 3.8/5; non-modified mean = 3.81/5) about their responses regardless of the Instruction Type and Adjective (exposed or new).^6^

The results support two predicted effects of the exposure items. First, participants reliably track talker-specific usage patterns of adjectives and choose the modifying talker for new instructions with previously observed adjectives (i.e., big/small). Second, participants generalized their assumptions to previously unobserved scalar adjectives and chose the modifying talker for instructions with new scalar adjectives. Thus listeners quickly adapt their expectations for a particular talker’s referring expressions.

However, these results are compatible with at least two classes of accounts. Participants could have inferred that one speaker provided the sufficient amount of information to uniquely refer, while the other did not (Informativity-based generalization). Alternatively, participants could have inferred that one of the speakers was more likely to produce modified utterances (Form-based generalization). In Experiment 2, we modified the instructions in the Generalization Phase to investigate which account better predicts listeners’ adaptation/generalization behavior.

## Experiment 2: Generalization from Under-Informative Evidence

### Experiment 2A: Informativity-Based vs. Form-Based Generalization for Talker-Specific Adaptation

Experiment 2A examined whether participants inferred that one of the speakers was more or less informative (Informativity-based generalization) or generalized based on utterance length (Form-based generalization). We replaced the bare noun instructions in the Generalization Phase of Experiment 1 with orthogonal color adjectives (e.g., *Click on the green car* when both cars in the scene are green). If generalization is based on informativity, participants should select the previously non-modifying (under-informative) speaker. If, however, generalizations are form-based (i.e., based solely on whether or not a speaker had used an adjective), participants should select the modifying speaker on both the color-adjective trials and the scalar adjective trials.

### Methods

#### Participants

Thirty-three English-speaking adults residing in the USA who had not previously participated in a study in this series were compensated $1.00 for taking part in the task on Amazon Mechanical Turk. We applied the same exclusion criteria as what we used in Experiment 1.

#### Materials

The visual and the audio materials for the Exposure Phase were identical to those used in Experiment 1. We constructed 12 new instructions for the Generalization Phase by replacing the non-modified instructions with instructions containing color adjectives. These instructions were paired with two-by-two grids with the contrastive item pair that differed in size along the same dimensions as the scalar adjectives used in the scalar modified trials, but did not differ in color. Thus, these color-modified instructions such as “*Click on the green bottle”* would not pick out a unique referent. The remaining 12 scalar-modified instructions such as “*Click on the wide bottle”* (**Figure 2**), carried over from Experiment 1, would pick out a unique referent. Thus, all instructions in the *Generalization Phase* contained either a scalar or a color adjective. Experiment 2A used the same instructions as Experiment 1.

#### Procedure

Procedure was identical to Experiment 1. Participants were not given feedback on their responses and asked to rate confidence in their selection after each item in the Generalization Phase.

### Results and Discussion

**FIGURE 4:**
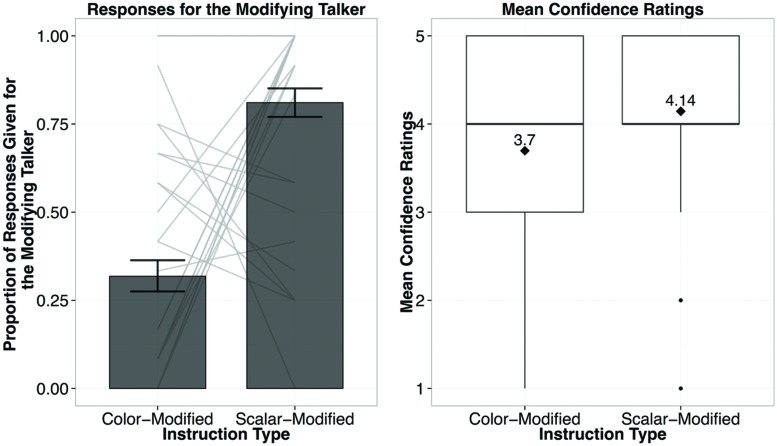
**Results from Experiment 2A, showing the proportion of responses given for the modifying talker by Instruction Type (left; light gray bars reflect individual participant means), and the confidence ratings for responses by Instruction Type (**right**; diamond dots reflect the mean rating out of 5)**.

Participants’ responses were similar to those in Experiment 1 (see **Figure 4**). For both observed and new scalar adjective types, they primarily picked the modifying speaker (81%). However, on the color-modified trials, participants showed preferences for the non-modifying talker (68%). These results show that participants are making informativity-based generalizations, choosing the previously non-modifying talker for modified yet under-informative instructions. The mixed-effects logistic regression found that the only reliable predictor of whether the listener chose the modifying speaker on a given trial was Instruction Type [scalar-modified (informative) vs. color-modified (under-informative); β = 6.428, *p* < 0.001].

In sum, these results suggest that not only have participants discovered that there is something linguistically different between the talkers, but also that one of these talkers was using pre-nominal modification to provide information that allows unique reference, whereas the other was not. Participants were willing to attribute new color-modified utterances to a talker they have never heard using color adjectives to modify. Thus participants have inferred that only one of the talkers uses modification to provide sufficient information for unique reference.

#### Comparison of Experiments 1 and 2A

We compared Experiments 1 and 2A using a mixed-effects logistic regression analysis with Experiment (1 vs. 2A), Adjective (exposed vs. new), and Instruction Type (under- vs. concisely-informative) as fixed effects, and subject and item as random effects, including by-subject (Adjective and Instruction Type) and by-item (Experiment and Instruction Type) random intercepts and slopes. We used a model with the correlations between the random slopes and random intercepts removed as recommended by Barr et al. (2013) when the maximal model fails to converge. We found evidence that is comparable to what we found in Experiment 1 for talker-specificity: Instruction Type was a predictor of the responses for the modifying talker, β = 5.752, *p* < 0.001. In addition, there was a predictive effect of Experiment (β = 0.807, *p* = 0.05). The predictive main effect of Experiment is likely driven by a smaller percentage of responses for the non-modifying speaker on the color-modified trials in Experiment 2A in comparison to the percentage of responses for the non-modifying speaker on the non-modified trials in Experiment 1.

To take a closer look at patterns of responses by individual participants, we plotted mean proportion of choice of the modifying talker with the light gray connecting bars in **Figure 4**. It is evident that there is a substantial amount of individual variation: while the majority of participants responded in a way that reflects informativity-based generalizations (lower responses for the modifying speaker on color-modified trials, and higher responses on the scalar-modified trials), some participants seem to be making form-based generalizations, as noted by relatively invariant responses for the modifying speaker across both trial types. We suspected that the individual differences stemmed from the fact that participants differed from each other in terms of their construal of the current task. Some might have assumed the goal of the task was to evaluate the clarity and helpfulness of the instructions, which would have encouraged them to focus on informativity of the instructions. Others might have tried to match instructions in terms of their formal similarity. To test this idea, we made this assumption explicit in our instructions to see whether that would affect patterns of participants’ responses.

### Experiment 2B: Effects of Adding a more Explicit Cue – Focus on Naturalness

In Experiment 2B, we repeated Experiment 2A but added an extra instruction that asked the listeners to pay close attention to potential speaker differences in “clarity” and “naturalness.” We hypothesized that the explicit instruction would increase informativity-based generalizations by highlighting the fact that instructions vary along the dimension of helpfulness in picking out a unique referent. We also report two follow-up analyses. First, we present a mixture model analysis of a combined dataset from Experiments 2A and 2B to further investigate the effect of explicit instructions. We then present data from a follow-up experiment in which comprehenders observed informative uses of color-adjectives and under-informative uses of scalar adjectives in the Generalization Phase. We predict a similar – possibly slightly diminished – degree of informativity-based generalization, which would rule out the possibility that the generalization is limited to a particular adjective type.

### Methods

#### Participants

Thirty-two English-speaking adults residing in the USA who had not previously participated in a study in this series were compensated $1.00 for taking part in the task on Amazon Mechanical Turk. We applied the same exclusion criteria as we used in the previous experiments.

#### Materials

Identical to Experiment 2A.

#### Procedure

The procedure was identical to Experiment 2A, except that participants received audio instructions instead of the written instructions used in Experiments 1 and 2A. This was to ensure that they heard all of the details of the instructions. Participants were told that the goal of the task is to select instructions by speakers that made the clearest or most natural instructions, and that at the end of the task there would be an opportunity for them to provide feedback on whether either of the speakers’ instructions were unusual in any way. At the end of the experiment participants were asked to indicate which speaker they thought was the clearest and most natural sounding, and then were asked to describe why they thought the other speaker was less clear or natural.^7^

### Results and Discussion

When attention was called to the clarity of the two speaker’s utterances, participants’ responses showed more pronounced trends toward informativity-based generalizations. Participants selected the talker who previously did not use adjectives in the Exposure Phase in the (under-informative) color-modified trials, 88% of the time. As in Experiment 2A, the only reliable predictor of whether the modifying talker was chosen was Instruction Type (β = 8.109, *p* < 0.001), meaning that yet again we see evidence for participants overall generalizing based on informativity.

#### Comparison of Experiments 2A and 2B

**FIGURE 5:**
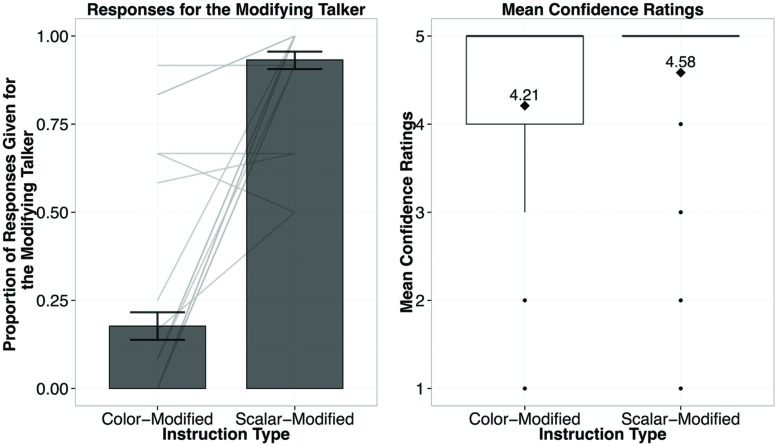
**Results from Experiment 2B, showing the proportion of responses given for the modifying talker by Instruction Type (left; light gray bars reflect individual participant means), and the confidence ratings for responses by Instruction Type (**right**; diamond dots reflect the mean rating out of 5)**.

We compared Experiments 2A and 2B using a mixed-effects logistic regression analysis with Experiment (2A vs. 2B), Adjective (exposed vs. new), and Instruction Type (scalar- vs. color-modified) as fixed effects, and subject and item as random effects, including by-subject (Adjective and Instruction Type) and by-item (Experiment and Instruction Type) random intercepts and slopes. We used a random-slopes only model as suggested by Barr et al. (2013), rather than a maximal model, or a model with independent random slopes and intercepts, as they both failed to converge. Instruction Type (β = 5.76*, p* < 0.001), the interaction of Experiment by Instruction Type (β = 2.888, *p* = 0.05), and a three-way interaction between the fixed effects (β = 2.366, *p* = 0.01) were significant predictors of whether or not participants chose the modifying speaker. The interaction of Instruction Type and Experiment supports the idea that explicit instructions biased participants to generalize more on informativity: with explicit instructions fewer participants attributed the color-modified instructions to the non-modifying (under-informative) talker, and fewer participants attributed the scalar-modified instructions to the modifying talker (see **Figure 5**). The explanation for the interactions will become clear in the following analyses.

#### Mixture Model Analysis of Experiments 2A and 2B

Because we hypothesized that explicit instructions would result in more informativity-based generalizations, we tested for patterns of generalization across participants. We did so by fitting multivariate mixture models to the data. Separate models were fit for each Instruction Type in each of the conditions for 1-6 components using the mixtools package (Benaglia et al., 2009) in R, which uses expectation maximization (EM) to estimate the optimal parameter values.

On the scalar-modified trials, participants primarily attributed these instructions to the modifying talker, and even more so in Experiment 2B. In Experiment 2A our mixture model analysis found that the majority (73%) of the participants selected the modifying talker for the scalar adjective trials on average 98.2% of the time, and the remaining 27% of participants selected the modifying talker on average 35% of the time. In Experiment 2B the model found that the majority (88%) of the participants selected the modifying talker for the scalar adjectives on average 98.2% of the time. The remaining 12% of the participants selected the modifying talker on average 59% of the time.

For color-modified trials, in Experiment 2A, a three-component model fit the data significantly better than the either the one-component [χ^2^(6) = 373.2, *p* < 0.001] or the two-component [χ^2^(3) = 18.8, *p* < 0.001] models. Participants responses fell into the following three categories: (1) 12% of the participants selected the modifying talker for these trials 98% of the time (evidence for form-based generalizations); (2) 30% of participants selecting the modifying talker 57% of the time (approximately chance-like behavior, indicating that they thought either speaker could have produced these instructions with equal likelihood); and (3) the remaining 58% of the participants picked the modifying talker only 5% of the time (evidence for informativity-based generalizations). In contrast, in Experiment 2B a two-component model fit the data significantly better than a one-component model [χ^2^(3) = 305.1, *p* < 0.001] or a three-component [χ^2^(3) = 0.14, *p* = 1] models. Individual participants responded in one of two ways: (1) 81.2% of the participants picked the modifying speaker only 5% of the time (evidence for informativity-based generalizations); and (2) the other 18.8% of the participants selected the modifying speaker for these trials 75% of the time (evidence for form-based generalizations).

**FIGURE 6:**
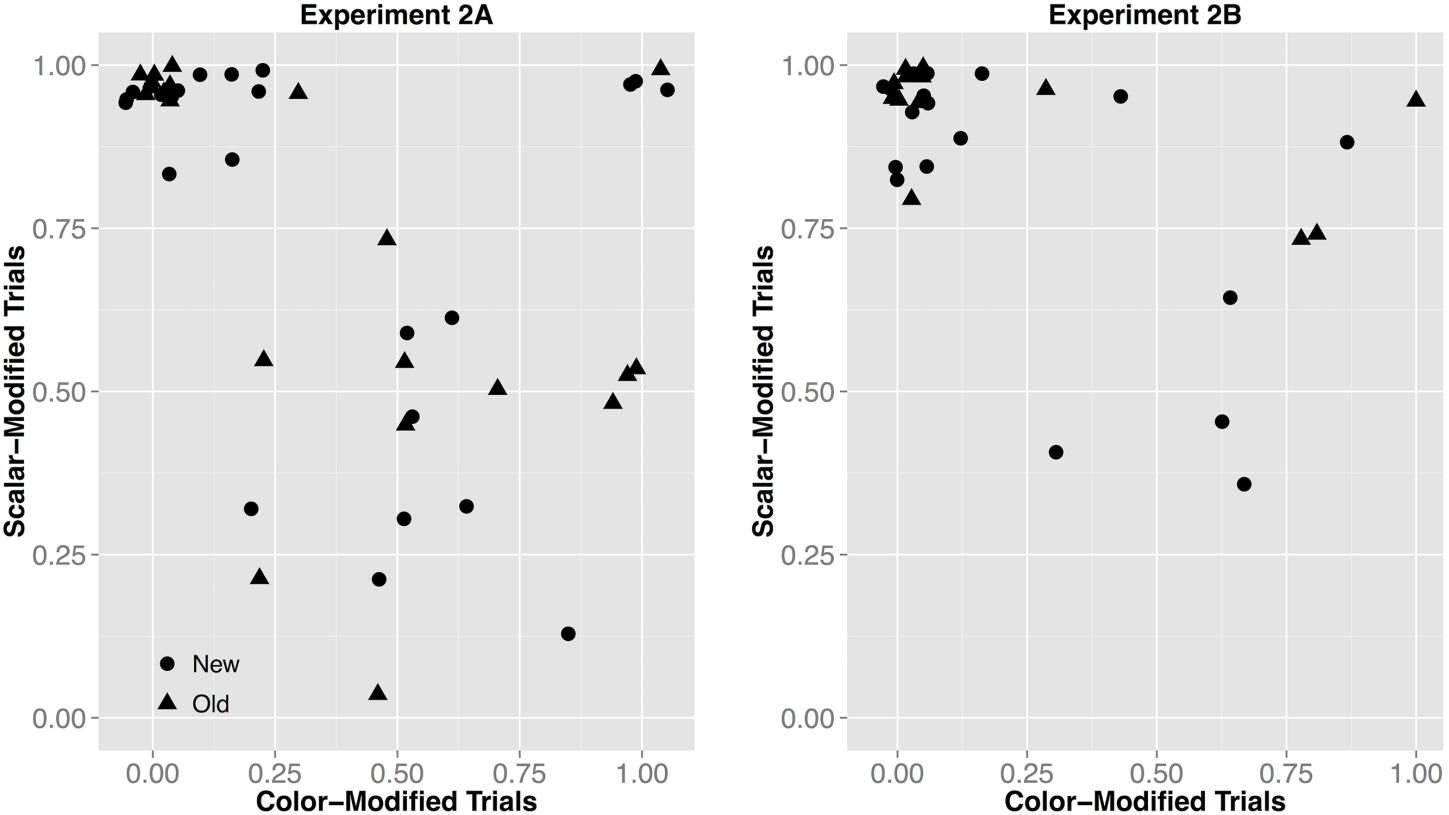
**Proportion of responses given for the modifying talker for color- by scalar-modified trials for each individual subject in Experiment 2A and Experiment 2B**. Informativity-based generalizations (dots expected to pattern in the top left) are observed when listeners primarily select the modifying talker for the scalar-modified trials (proportion approaching 1.0) and rarely for the color-modified trials (proportion approaching 0.0). Form-based generalizations (dots expected to pattern in the top right) are observed when listeners primarily select the modifying talker for both trials (proportions approaching 1.0).

This analysis reveals that there is more variability in participant response patterns in Experiment 2A, compared to Experiment 2B. This can be seen in the tighter clustering pattern toward the top left corner in Experiment 2B in **Figure 6**. If listeners generalizations are informativity-based, we expect results to cluster in the top left (meaning that an individual always picked the modifying speaker for the scalar-modified trials, thus approaching 1, and almost never for the color-modified trials, approaching 0), whereas if they were form-based we expect clustering in the top right corner (where the proportion of responses for the modifying speaker is near 1 for both instruction types). In sum, calling attention to the quality of the instructions made listeners more willing to infer that the non-modifying speaker would be less pragmatically optimal and therefore *more* likely to use an under-informative color-adjective.

#### Ruling out an Alternative Explanation based on Adjective Class

One possible concern is that the results in Experiments 2A and 2B could be due to listeners’ tendency to associate a particular talker with a particular adjective type. Participants might have assumed that one of the talkers liked to use scalar adjectives and the other non-scalar adjectives. While associations like these are not attested in any previous research, it is possible that participants in the current study might have inferred that, at the very least, one of the speakers was more likely to use scalar adjectives than the other. To rule out this possibility, we conducted an additional version of Experiment 2 (*n* = 32) in which the items in the Generalization Phase contrasted in color rather than a scalar dimension. That is, in contrast to Experiments 2A and 2B, color-adjectives in the Generalization Phase were helpful in selecting a unique referent whereas scalar adjectives were not. If participants are generalizing based on informativity they should attribute the contrastive, color-modified instructions to the modifying speaker.

As in Experiments 2A and 2B, we found that the only reliable predictor of whether participants selected the modifying speaker was Instruction Type (β = 6.459, *p* = 0.05). Participants selected the modifying speaker for the color-modified informative instructions 84% of the time. Participants also selected the modifying speaker for the non-informative scalar-modified instructions. However, as we predicted, they did so less than for the color-modified instruction (58% compared to 84%).

What accounts for the relatively high selection rate (58%) of the previously modifying (informative) talker for the under-informative scalar-modified instructions? We have two hypotheses. First, listeners have weighted heavily their direct observation of one talker using scalar adjectives in the Exposure Phase. This might have made it difficult for them to inhibit the expectation that the previously modifying talker would continue to use scalar adjectives. Second, unlike color-modifiers, talkers in general rarely produce scalar-modifiers in non-contrastive situations (Pechmann, 1989; Belke and Meyer, 2002; Sedivy, 2003; Brown-Schmidt and Tanenhaus, 2008; Viethen et al., 2012). Therefore, a listener would not expect a speaker who did not use adjectives at all to begin producing a non-contrastive scalar-modified utterance compared to a non-contrastive color-modified utterance. In sum, these results provide additional support for our claim that participants were paying attention to the informativity of the talkers. As the same time, participants may have different expectations for different classes of adjectives (e.g., scalar- vs. color-adjectives) in terms of how reliably they would support an informativity-based generalization.

## Experiment 3: Talker-Specific Adaptation with Over-Informative Evidence

As we noted earlier, speakers rarely under-modify (except in highly collaborative tasks; Brown-Schmidt and Tanenhaus, 2008). Do listeners’ prior beliefs based on general statistics like these have any influence on ways in which they adapt their referential expectations? If so, how? As we mentioned in the introduction, the prevalent over-modification observed in natural discourses in contexts like the ones we used should lead to more conservative generalization compared to cases of under-modification. In particular, listeners might consider that a single instance of a redundant adjective use is not a good predictor of the same speaker’s future pragmatic language use.

Integration of prior likelihoods into statistical inferences has proven effective in generalizing information meaningfully based on a limited amount of the input. For instance, word learners generalize information about novel word-referent mappings (e.g., “blicket” for a novel object) based on their prior beliefs about how nouns are used, who provided the data, and how the evidence is sampled (Xu and Tenenbaum, 2007). They then make inferences about how readily an observed word-referent mapping should be generalizable to other referents of the same kind rather than being restricted to a unique individual or property. Thus, by integrating relevant prior beliefs, listeners are able to evaluate the input with respect to how reliably it can predict previously unseen data, which helps reduce the chance of over- or under-generalization due to over-fitting their expectations to data observed locally.

### Methods

#### Participants

Thirty-two English-speaking adults residing in the USA who had not previously participated in a study in this series were compensated $1.00 for taking part in the task on Amazon Mechanical Turk. We applied the same exclusion criteria as what we used in previous experiments. An additional participant completed the task but was excluded from the analysis for giving incorrect responses during the Exposure Phase.

#### Materials

Audio stimuli for the Exposure Phase (20 items) were identical to those in Experiments 1 and 2. Visual stimuli were modified so that there was no size contrast pair and each two-by-two grid consisted of four singleton images. This manipulation rendered a non-modifying instruction to be concisely informative (e.g., “Click on the cake”), and a modified instruction to be over-informative (e.g., “Click on the big cake”). Visual stimuli in the Generalization Phase (24 items) were identical to those in Experiments 1 and 2, containing a visual contrast pair. 12 of the 24 items were associated with a single-modified instruction (e.g., “Click on the wide bottle”) and the rest were associated with a double-modified instruction with both an informative scalar adjective and a redundant color adjective (e.g., “Click on the wide green bottle”). We predicted that if listeners were generalizing on informativity, rather than form, that they should attribute the single-modified (concisely informative) instructions to the previously non-modifying speaker, and the double-modified instructions (over-informative) to the modifying speaker, who appears to be habitually over-informative.

#### Procedure

As in the previous experiments, participants completed all the 20 exposure trials and 24 generalization trials consecutively. Participants read the same instructions used in Experiments 1 and 2A. Participants rated their confidence on each trial.

### Results and Discussion

**FIGURE 7:**
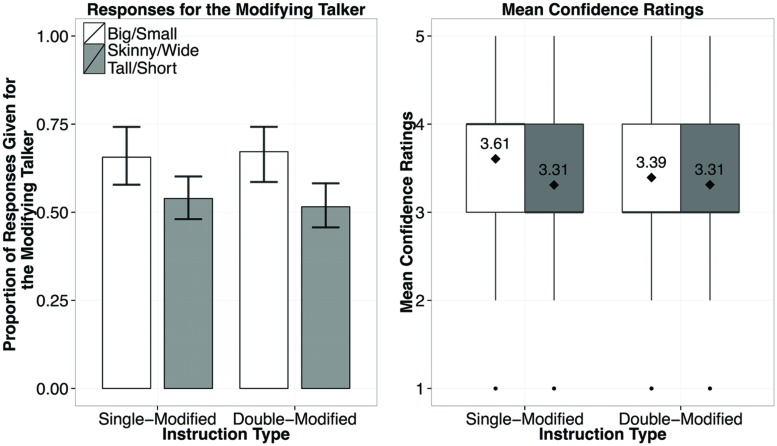
**Results from Experiment 3, showing the proportion of responses given for the modifying speaker by Instruction Type (left), and the confidence ratings for responses by Instruction Type (right; diamond dots reflect the mean rating out of 5)**.

In contrast to the cases with an under-modifying talker (i.e., Experiments 1 and 2), the results from Experiment 3 show no clear evidence for informativity-based generalization (**Figure 7**). In a mixed-effects logistic regression Instruction Type (concise-or over-modification) was not a reliable predictor (*p* > 0.1), nor was the interaction of Instruction Type and Adjective (*p* > 0.1). The only reliable predictor of when participants would choose the modifying (over-informative) speaker was whether a previously encountered or a new scalar adjective was used (β = 0.959, *p* = 0.05): participants were more likely to choose the modifying speaker if a previously exposed adjective (*big* or *small*) was used (67%) regardless of whether it was used with a color adjective. For the new adjectives, there was no clear trend for participants to attribute the use of the new adjectives to either speaker, attributing them equally to both speakers (choosing the modifying speaker in for 52% of the responses).

As predicted, we found an asymmetry between the cases of under-modification and over-modification, in which listeners do not seem to make talker-specific informativity-based generalizations from exposure to over-modified instructions. This null effect with over-informative input was, however, somewhat surprising given the reliable effects of talker informativity found in Experiments 1 and 2. Before we conclude that this pattern of results is due to participants being more conservative about generalizing from over-informative utterances, we need to rule out another possibility. Perhaps participants did not notice that one of the talkers was over-modifying in the Exposure Phase.

#### Ruling out an Alternative Explanation based on a Failure to Generalize overall for Over-Informative Utterances

Unlike the under-modifying instructions used in Experiments 1 and 2, over-modifying instructions do not create referential ambiguity. Thus, if talker-specific adaptation requires an observation of a clear “error” signal based on possible miscommunication, then the manipulation we used might have been too subtle to trigger adaptation, To address this possibility we conducted a follow-up experiment (*n* = 32), modeled on Experiment 1 to see if we could observe form-based generalizations. Participants observed the same two speakers describing images from a two-by-two grid that was comprised entirely of unrelated singleton images (as in Experiment 3) in both the Exposure Phase and the Generalization Phase. Thus, in both the Exposure Phase and the Generalization Phase modified instructions were over-informative, and non-modified instructions were concisely informative. The results demonstrated that participants were more willing to attribute the over-modified utterances to the modifying speaker (85% compared to 18% for the non-modified), regardless of the adjective used (β = 10.27, *p* < 0.001). This makes it unlikely that participants in Experiment 3 were simply not aware of talker-differences in the Exposure Phase. It is, however, still possible that they did not regard over-informative utterances to be communicatively sub-optimal because they did not cause any referential ambiguity.

To see whether this might be the case for our instruction, we looked at responses in the follow-up questionnaire in Experiment 3 (identical to that of Experiment 2B), which asked participants to comments on the clarity and naturalness of the two talkers’ instructions. Participants were divided as to which talker they preferred: some participants found the over-modifying talker to be clearer and more helpful (23%); others considered the over-modifying talker to be redundant and potentially confusing (45%). The remaining participants commented on the quality of the speakers’ voices, the recordings, or gave no response (32%). Thus the asymmetrical treatment of under- and over-modifying utterances could be due in part to listeners not considering over-modifying utterances to be communicatively sub-optimal. This would make it less likely for them to expect similar behavior from the same talker across different contexts. In Experiment 4, we manipulated the Exposure phase of Experiment 3 to highlight the fact that producing over-modifying instructions can, at least in some cases, hinder referential communication.

## Experiment 4: Drawing More Attention to the Fact that Over-Informative Information is Not Helpful

In Experiment 3, and in previous research (Engelhardt et al., 2006; Arts et al., 2011), listeners have been shown to treat some instances of over-specification as facilitatory. In Experiment 4, we introduced two modifications to the paradigm used in Experiment 3, with the intention of highlighting the potential pitfalls of over-modification in the current referential task. First, we truncated 50% of the audio instructions such that the concise referential expressions communicated sufficient information for unique referent identification (e.g., “Click on the ca-” when a target is “camera”) whereas the over-modified expressions do not (e.g., *“Click on the sma-”* when there is more than one small referent in a visual scene). Second, after each trial, we provided feedback identifying the talker’s intended referent. We implemented these changes to emphasize the fact that producing a superfluous adjective can result in referential ambiguity.

### Methods

#### Participants

Thirty-four English-speaking adults residing in the USA who had not previously participated in a study in this series were compensated $1.00 for taking part in the task on Amazon Mechanical Turk. We applied the same exclusion criteria as what we used in the previous experiments.

#### Materials

Visual and audio stimuli were identical to Experiment 3 except for the following three changes in the Exposure Phase. First, the audio instructions were truncated mid way: five out of the 10 unmodified instructions were cut off after the onset syllable of the noun (e.g., “Click on the ca-” when a target is “camera”), the remainder were truncated mid-word after the second consonant (e.g., *“Click on the cam-”*). Five of the 10 modified instructions were truncated after the adjective (e.g., *“Click on the small”* when there is more than one small referent in a visual scene), and the remaining modified instructions were truncated after the onset syllable of the noun (e.g., *“Click on the small ca-”*)^8^.

**FIGURE 8:**
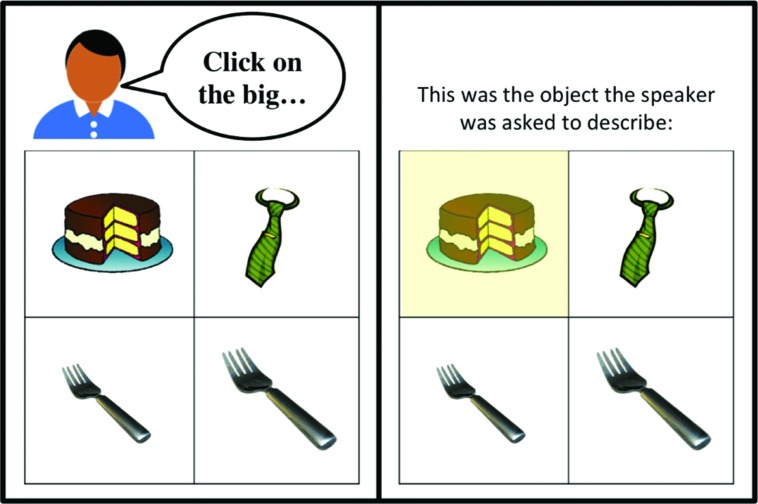
**An example of a trial (audio instructions are shown here in speech bubbles) with the modifying speaker (left) in the Exposure Phase in Experiment 4**. Participants clicked on the image in the grid to respond. After each trial participants were shown which of the four images the speaker was asked to describe **(right)**.

Secondly, half of the Exposure Phase trials contained two-by-two grids with a contrast pair, and half contained four singleton items. Crucially the instructions produced by both speakers never referred to an item from the contrasting pair. Third, after each trial participants were shown which item the speaker was originally asked to describe. On the trials where the recording was cut off after the adjective, it was expected that the use of the redundant scalar adjective would be seen as misleading. An example trial from Experiment 4 can be seen in **Figure 8**. The Generalization Phase was identical to that of Experiment 3.

#### Procedure

The procedure was the same as in Experiment 3.

### Results and Discussion

**FIGURE 9:**
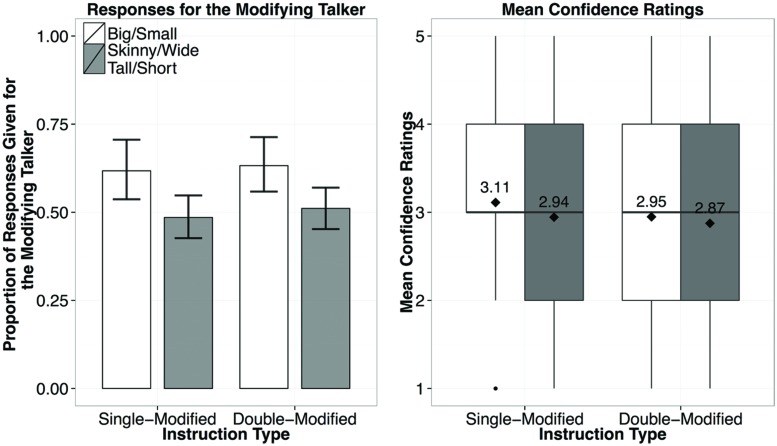
**Results from Experiment 4, showing the proportion of responses given for the modifying speaker by Instruction Type (left), and the confidence ratings for responses by Instruction Type (right; diamond dots reflect the mean rating out of 5)**.

Despite the changes we made to the Exposure Phase, the results were nearly identical to those in Experiment 3 (**Figure 9**). In a mixed-effects logistic regression Instruction Type was not a significant predictor of whether participants chose the modifying speaker (β = 0.169, *p* > 0.1), However, whether an exposed or a new adjective was used was a significant predictor (β = 0.635, *p* = 0.05). Participants were overall more likely to attribute the instructions containing the words *big* or *small* to the modifying speaker (62%) than the instructions containing new adjectives (50%), regardless of the Instruction Type (single modified, or double-modified, as noted by the lack of an interaction predictor). The results of Experiment 4 strongly suggest that the absence of evidence for generalization in a talker-specific manner from over-informative evidence is not due to listeners failing to register its sub-optimality in the communicative task at hand. It is more likely that listeners did not weigh evidence of over-informative instructions as much as that of under-informative ones, leading to more conservative generalization of their referential expectations at the talker level.

## General Discussion

We proposed that listeners adjust their expectations for how individual talkers might vary in their uses of referring expressions. This permits listeners to maintain the assumption that talkers are generally rational, rather than only sometimes rational, while allowing them to flexibly cope with the variability in speakers’ referring expressions. We presented four sets of talker-selection experiments, examining if, and, if so, how, listeners adapt and generalize their referential expectations according to the observed input. We examined cases in which one of the two talkers produced either an under-modified or an over-modified utterance for a referent in a visually co-present context.

### Summary of Results and Contribution

#### Under-Modification

With under-modified instructions, we found clear evidence that listeners adapted to talker-specific differences in the use of pre-nominal adjectives along the dimension of informativity. When a talker under-modified, listeners inferred that that talker would not formulate an informative utterance with other scalar adjectives (Experiments 1 and 2). Moreover, they considered the possibility that the same talker would formulate under-informative utterances with color adjectives (Experiment 2). This demonstrates that listeners generalized the information given in the Exposure Phase based on informativity. Importantly, we found evidence for informativity-based generalizations even when the evidence was implicit. However, the proportion of informativity-based generalizations increased when the instructions directed participants to pay attention to the clarity of the talkers’ instructions.

Our results with under-modified instructions build on Grodner and Sedivy’s (2011) results in three ways. First, Grodner and Sedivy reported that talker-specific modulation of pragmatic processing, in their paradigm, required explicit top–down information about the speaker being pragmatically impaired (though they mention that there was still a trend when top–down information was not provided). In contrast, our experiments with under-modification induced talker-specific adaptation of referential expectation without such an explicit instruction. This suggests that listeners are in principle capable of modulating their expectations based on bottom–up input alone. Second, our results contrasted generalizations based on under- and over-informative utterances, and thereby shed light on the dimensions over which listeners are generalization. Third, we show that, depending on the participant and the task, generalization can be more or less form-based and informativity-based.

#### Over-Modification

With a speaker who regularly over-modifies, we did not observe informativity-based generalization (Experiment 3). Even when a superfluous modifier was clearly unhelpful in reference resolution, listeners did not assume that overly informative utterances were a characteristic of an individual talker (Experiment 4). Listeners did make talker-specific generalizations, but they were overwhelmingly form-based rather than informativity-based. Our results with over-modified instructions might seem superficially inconsistent with Grodner and Sedivy’s evidence for talker-specific adaptation with over-modified instructions. Recall, however, that some researchers (Arnold et al., 2007; Grodner and Sedivy, 2011) noted that they obtained robust results only when they explicitly called attention to the speaker’s overall linguistic incompetence. Thus our results can be viewed, akin to the findings of Grodner and Sedivy, as supporting the suggestion that generalization from over-modification is strongest when there is top–down information that establishes a causal link between the redundant use of a prenominal modifier and the pragmatic propensities, or even, the linguistic competence, of the talker.

The asymmetry between the results with under-modification and over-modification is particularly striking. It provides strong support for the assumption that generalization takes into account prior beliefs based on the statistical structure of the data, in this case typical patterns of modification. We discussed the possibility that informativity-based generalizations might be weaker with over-modification than with under-modification because under-modifying utterances interfere more with communication in the task at hand. Admittedly, under-modifying utterances do not allow listeners to single out a unique referent, which calls attention to the sub-optimality of those utterances. In contrast, over-modifying utterances, allow listeners to pick out an intended referent. In fact, providing additional information is often considered a sign of helpfulness (Engelhardt et al., 2006; Arts et al., 2011). The likelihood of communicative error in a given context cannot, however, account for our pattern of results. The truncated utterances in Experiment 4 created referential ambiguity, drawing attention to the fact that by including superfluous material the over-modifying talker generated referring expressions that resulted in communication failure.

It is possible that general inferences about informativity from over-modification might emerge only with more robust manipulations in highly collaborative tasks, e.g., in a video game task where timely actions based on communication with a partner are required. Alternatively, because oﬄine measures do not capture real-time expectations, an online measure might reveal effects that are not captured in oﬄine measures, e.g., reaction times (Engelhardt et al., 2011), or eye-tracking (Grodner and Sedivy, 2011). We leave the question of under what conditions, if any, listeners might make informativity generalizations based on over-modification as an issue for future research. Nonetheless our results clearly demonstrate that prior beliefs of the listener about characteristics of referring expressions, and not just the optimality of an utterance with respect to a particular context, are an important factor for understanding reference generation and understanding. Along these lines, it will be important in future research to further investigate reasons why speakers might include more information in a referential expression than is strictly necessary for identifying a referent (for discussion see Isaacs and Clark, 1987; also Heller et al., 2012; Gorman et al., 2013; Gegg-Harrison and Tanenhaus, in review).

We began by considering how listeners might make rational use of linguistic information despite the fact that the linguistic input often includes more or less information than what is necessary and sufficient for a given referential intention. The current results help provide a critical piece of the puzzle: listeners can flexibly adapt their estimates of an expected amount of information associated with the given referential intention. Our findings demonstrate that the process of adaptation includes statistical inferences. Those inferences are conditioned on factors such as types of evidence (under- and over-informative), classes of adjectives, and listeners’ prior beliefs about how reliably a particular type of non-optimal utterance would convey information about whether the talker would be non-optimal in the future.

#### Individual Differences

Although it was not a main focus of our study, the results in Experiments 2A and 2B revealed clear individual differences among participants with regard to their construal of the referential task. In Experiment 2B, we used an identical set of visual and audio stimuli as in Experiment 2A while drawing participants’ attention to the fact that this is a task about evaluating the quality of instructions produced by two individual talkers. This manipulation made participants’ responses significantly more uniform such that a larger proportion of participants provided responses that indicated informativity-based, rather than form-based, generalization. This suggests that participants vary in their construal of a task, a context, and a goal of referential communication even in a simple paradigm like the one we used in our study (for individual differences in semantic and pragmatic interpretations of utterances, see Noveck and Posada, 2003; Bott and Noveck, 2004; Degen and Tanenhaus, 2015; Yildirim et al., 2016). Importantly, participants’ assumptions about the task can determine the dimensions along which they generalize (see Brown-Schmidt and Fraundorf (2015) for evidence that perceived interaction influences use of common ground information).

### Future Directions

The differences among participants suggest that one fruitful direction for future research will be to look at various contextual factors that likely influence the process of speaker-specific generalization of referential expectations. We mentioned in the introduction that studies on phonetic adaptation and generalization revealed that listeners structure their knowledge with respect to talker groups and situations. For instance, listeners do not *indiscriminately* generalize their knowledge about one talker’s speech categories to a different talker, but facilitation after exposure to multiple talkers with the same foreign accent generalizes to new speakers with the same or similar accents (Bradlow and Bent, 2008). Similarly, listeners may be able to structure their expectations for referential expressions according to speaker groups or conversational contexts. For instance, adult speakers may produce more redundant modifiers when talking to a young child compared to when talking to another adult (e.g., *Look at the big brown doggy*! when there is only one dog in sight). Integrating contextual factors like this would help listeners “explain away” some of the variability observed within a speaker and further reduces the risk of under- or over-generalization.

We believe that our results have implications for research in reference production, including reference expression generation models (REG models). Models to date appear to take into account some manner of contextual information, primarily including referents that are visually or linguistically present in the context (see Krahmer and van Deemter, 2012, for a survey of work in REG to date). Some models attempt to accommodate interlocutor-specific information (e.g., Heeman and Hirst, 1995; Jordan and Walker, 2005) by producing referential expressions that reflect conceptual pact information (referential expressions that have been negotiated between particular interlocutors, see Clark and Wilkes-Gibbs, 1986; Brennan and Clark, 1996). We propose that future models of reference production should also take into account how interlocutors negotiate their referential expressions to find the most optimal level of reference given their certainty about the contextual and mutually shared information. In particular, such models should examine how these expectations might change over time given both the evidence at hand, and the interlocutors’ prior beliefs.

Another fruitful line of research is examining how children treat under- and over-modifying utterances and whether they adapt their referential expectations in a talker-specific manner. Previous studies have reported that preschoolers can discriminate talkers’ pragmatic abilities (e.g., Koenig and Harris, 2005; Scofield and Behrend, 2008), based on utterances with clear errors (e.g., using “key” to refer to a ball). It is, however, yet to be clear whether they can distinguish talkers based on the *quantity* of information provided (Eskritt et al., 2008). We have conducted a preliminary study using a paradigm similar to Experiment 1 in the current paper. We found that preschoolers, unlike adults, have difficulty associating under-modifying utterances with an individual talker (Pogue et al., 2015). This may be due to a number of possible reasons including their limited memory and attention span, general insensitivity to pragmatic principles in conversation and weaker assumptions for across-talker variability. Further investigation, both oﬄine judgment studies like ours as well as online eye-movement studies, is necessary to paint a complete picture of the developmental trajectory of the ability to derive referential expectations.

Finally, our results open up a number of questions as to what is intended by *informativity*. As we discussed in the introduction, most theories so far have defined informativity as an expected amount of information with respect to an array of referents in a visual scene. Anything that exceeds the amount is considered over-informative and anything that falls short of it is considered under-informative. And these deviations are expected to trigger pragmatic inferences. Our results, however, yield strong support for the view that what counts as informative can change depending on a talker and a context. Listeners constantly update their expectations as they gain more information about the talker and the context. Future studies on informativity should therefore explore processes in which the speaker and the listener negotiate means and a context of reference, reducing uncertainty regarding form-referent mappings in a collaborative dialog.

## Author Contributions

AP, CK, and MT designed the research, AP performed the research, AP analyzed the data and drafted the paper. CK and MT provided feedback and edited AP’s drafts.

## Conflict of Interest Statement

The authors declare that the research was conducted in the absence of any commercial or financial relationships that could be construed as a potential conflict of interest. The reviewer and handling Editor declared their shared affiliation, and the handling Editor states that the process nevertheless met the standards of a fair and objective review.

## Footnotes

1 Our comments apply only to how people weight evidence that is presented verbally, as in the Linda problem. Many of the Tversky and Kahneman heuristics that lead to fallacies are based on the people’s priors being distorted, e.g., overestimating the likelihood of events like airplane crashes, children being kidnapped, and infection from Ebola because the input, e.g., news coverage, is distorted. Thus people might make judgments and decisions that are objectively incorrect, even if their reasoning followed the principles of rational inference.

2 We chose to refer to the talkers modifying and non-modifying for the following two reasons. First, and most crucially, modifying/non-modifying and over-/under-informative are two orthogonal dimensions. A modified utterance, e.g., “Click on the big apple,” can be over- or under-informative given a visual scene and comprehenders’ construal of the task. (Similarly, in Experiment 2, a color-modified utterance is modified but under-informative.) In our experiments the two talkers are distinguished by the forms of the utterances they use. Participants could either attribute the difference between the talkers to differences in informativity or to preferences in utterance length. Therefore, it is more appropriate to use modifying and non-modifying to refer to the two talkers. Secondly, referring to the talkers as modifying/non-modifying provides a coherent thread across all of the experiments. With our current manipulation, the input sentences in the exposure phase are identical across experiment (i.e., one talker produces modification and the other does not). Whether these utterances are informative or not differs depending on the visual context (i.e., experiment).

3 All of the experiments discussed in this paper were carried out in accordance with the recommendations of the Research Subjects Review Board at the University of Rochester, all participants gave consent to participate in the studies. They were given a digital copy of the consent form, and were asked to click a radio button labeled “I accept” to indicate that they had read and understood the consent form. All participants gave informed consent in accordance with the Declaration of Helsinki.

4 Amazon’s Mechanical Turk permits requesters to set selection criteria, and only the Turkers that meet the selection criteria are shown the task in their list of available HITs (Human Intelligence Tasks). For the purposes of this task we set the selection criteria to include a location restriction to users in the USA, who have an overall approval rate of 95% or higher, and who have not participated in any of the other experiments related to those described in this paper. Participants were told in the description of the task that we were looking only for native English speakers (having learned English at the age of five or younger). Participants were additionally excluded if they gave two or more incorrect responses on the modified Exposure Phase (EP) trials in Experiments 1 and 2, on either type of EP trial in Experiment 3, or non-modified EP trial in Experiment 4. Participants who viewed the HIT but did not complete the task were excluded from the analyses, and are not reported here.

5 The same factors were used in a mixed-effects logistical regression for all experiments unless noted otherwise.

6 In this and all of the other experiments we report below, the main effects (Instruction Type and Adjective) were not a significant predictor of the confidence rating scores. Therefore, we do not discuss these results in subsequent experiments; instead we report the data in the Figures.

7 The majority of the respondents indicated that that they thought that the less clear speaker did not provide necessary adjectives / enough information to help pick out objects, only four of the 32 respondents indicated that they thought one of the speakers was less clear or natural for other reasons, such as the quality of the speaker’s voice (*n* = 2), or thought they were both quite clear (*n* = 2). We take this as evidence that the participants overall as a group interpreted the instructions to be about the informativity of the instructions rather than features of the speakers’ voices.

8 We chose this manipulation over more overt ways of highlighting over-informativity of the modifying-talker’s utterances such as saying “This speaker has only four words to spend” for the following two reasons. First, we were concerned that a word limit (“This speaker has up to four words to spend”) would make the task highly unnatural. We could not find a naturalistic context in which a speaker needs to control the number of words in a spoken sentence and we did not know how our participants would construe a situation like that. Second, if we introduced a word or a time limit in the introduction, listeners would likely expect the speaker to alter the syntactic structure of their instruction (e.g., “big apple” rather than “Click on a big apple”) and/or increase their speech rate. However, including modulations like these would introduce other unknown factors such as (un)intelligibility of instructions, which would make the experiment less comparable to the other experiments reported in the current paper. For these reasons, we used the cover-story, which supports the assumption that the speakers were unaware of this problem and hence their syntactic and phonological formulations of instructions were consistent with those in other experiments.
